# Analysis of Hand Joint Space Morphology in Women and Men with Hereditary Hemochromatosis

**DOI:** 10.1007/s00223-022-01050-3

**Published:** 2023-02-04

**Authors:** Ursula Heilmeier, Andrew J. Burghardt, Justin J. Tse, Puneet Kapoor, Kathryn S. Stok, Sarah Manske, Reinhard E. Voll, Georg Schett, Stephanie Finzel

**Affiliations:** 1 Department of Rheumatology and Clinical Immunology, Faculty of Medicine, University of Freiburg - Medical Center, Hugstetterstraße 55, 79106 Freiburg, Germany; 2 Musculoskeletal Quantitative Imaging Research Group, University of California San Francisco, 185 Berry Street, San Francisco, CA 94158 USA; 3 Department of Radiology and Biomedical Imaging, University of California San Francisco, 185 Berry Street, San Francisco, CA 94158 USA; 4 Department of Radiology, Cumming School of Medicine, McCaig Institute for Bone and Joint Health, University of Calgary, 3280 Hospital Drive NW, Calgary, AB T2N 4Z6 Canada; 5 Department of Biomedical Engineering, The University of Melbourne, Parkville, VIC Australia; 6 Department of Internal Medicine 3, Rheumatology and Immunology, Friedrich-Alexander-University Erlangen-Nürnberg (FAU) and Universitätsklinikum Erlangen, Erlangen, Germany

**Keywords:** Hereditary hemochromatosis, Hemochromatosis arthropathy, High-resolution peripheral computed tomography, Joint space

## Abstract

Hereditary hemochromatosis (HH) causes unbalanced iron deposition in many organs including the joints leading to severe cartilage loss and bone damage in the metacarpophalangeal joints (MCPJ). High-resolution peripheral quantitative computed tomography (HR-pQCT) and its joint space width (JSW) quantification algorithm quantifies *in vivo* 3D joint morphology. We therefore aimed to (i) determine feasibility and performance of the JSW algorithm in HH, (ii) quantify joint space morphology, and (iii) investigate the relationship between morphological and clinical parameters in HH. Here, we performed an exploratory study on 24 HH patients and sex- and age-matched controls using HR-pQCT imaging of MCPJ. Mineralized bone structure was automatically segmented from the grayscale image data and periosteal surface bone masks and joint space masks were generated. Mean, minimal, and maximal joint space width (JSW; JSW.MIN; JSW.MAX), JSW heterogeneity (JSW.SD), JSW asymmetry (JSW.AS), and joint space volume (JSV) were computed. Demographics and, for HH patients, disease-specific parameters were recorded. Segmentation of JS was very good with 79.7% of MCPJs successfully segmented at first attempt and 20.3% requiring semi-manual correction. HH men showed larger JSV at all MCPs (+ 25.4% < JSV <  + 41.8%, *p* < 0.05), larger JSW.MAX at MCP 3-4 (+ 14%, 0.006 < *p* < 0.062), and wider JSW (+ 13%, *p* = 0.043) at MCP 4 relative to HH women. Compared to controls, both HH men and HH women showed larger JSW.AS and smaller JSW.MIN at all MCP levels, reaching significance for HH men at MCP 2 and 3 (JSW.AS: + 323% < JSW.AS <  + 359%, 0.020 < *p* < 0.043; JSW.MIN: − 216% < JSW.MIN < − 225%, *p* < 0.043), and for women at MCP 3 (JSW.AS: + 180%, *p* = 0.025; JSW.MIN: − 41.8%, *p* = 0.022). Time since HH diagnosis was correlated positively with MCP 4 JSW.AS and JSW.SD (0.463 < *ρ* < 0.499, *p* < 0.040), and the number of phlebotomies since diagnosis was correlated with JSW.SD at all MCPs (0.432 < *ρ* < 0.535, *p* < 0.050). HR-pQCT-based JSW quantification in MCPJ of HH patients is feasible, performs well even in narrow JS, and allows to define the microstructural joint burden of HH.

**Supplementary Information:**

The online version contains supplementary material available at 10.1007/s00223-022-01050-3.

## Introduction

Hereditary hemochromatosis (HH) is a autosomal recessive disease associated with increased intestinal iron absorption and pathological iron deposition with progressive organ damage if left untreated [1]. To date, at least five different mutations have been identified that may result in HH with different penetrance. These include a mutation in the hemochromatosis gene HFE causing the so-called HFE-associated hereditary hemochromatosis, which is most commonly found in individuals of European ancestry [2], and mutations in the hemojuvelin (HJV) or hepcidin gene (HAMP) causing juvenile HH [3]. In addition, mutations in the transferrin receptor 2 gene and in the metal-transporter ferroportin (FPN1) gene have been identified resulting in TfR2-related HH or ferroportin disease, respectively [4] [5].

While excess iron deposition in the liver and consecutive liver cirrhosis and cancer are considered the leading causes of mortality in HH patients [6], it is the HH-related musculoskeletal manifestations and particularly arthralgias and HH arthropathy which often impact patients´ quality of life [7, 8] [9] [10] and which are common presenting features of HH disease [11] [12] [13]. Studies have shown that in 11% to 57% of HH patients, it was arthralgias (typically of the second or third metacarpophalangeal joint) which brought HH patients to seek initial medical care and led to HH diagnosis [14] [15]. Similarly, radiographic arthropathy in HH patients has been detected in 37% to up to 81% of HH patients [16] [17] and was also seen even in 33% of asymptomatic HH patients [15]. In this light, radiographic HH arthropathy does commonly occur as a presenting feature in both asymptomatic and symptomatic HH patients and should alert physicians of the diagnosis of HH and prompt them to initiate further HH testing. Given that arthropathy can predate HH diagnosis for up to 10 years [10] [18] [14] [19] and was also reported in asymptomatic HH patients where organ damage can still be prevented by early start of phlebotomy therapy, it is of major importance and of prognostic relevance [20] that HH arthropathy is detected as early and as reliably as possible. This would allow asymptomatic HH patients to get started on phlebotomy early and help prevent organ damage to other organs such as liver, heart, or pancreas to occur. In HH patients presenting with arthralgias, early quantification of HH joint damage may help shorten the time to HH diagnosis and thus help to start patients as early and fast as possible on phlebotomy treatment which –although not suited to reverse or ease HH arthropathy or arthralgias [9]—has proven effective in preventing, slowing, and reversing iron overload-induced damage to other organs such as the liver and also in reducing HH-related mortality [11].

While the pathophysiology underlying HH arthropathy is still widely unknown [21], the current gold standard for detecting and monitoring HH-related impact on the joints is considered conventional radiography [22]. However, given the very narrow joint spaces associated with HH, conventional radiography as a 2D technique with a limited image resolution up to maximally 0.3 mm may [23] underestimate the structural changes in the joints of HH patients. Subtle HH arthropathic changes may thus be missed risking delay of HH diagnosis. Therefore, novel three-dimensional imaging techniques with higher resolution may help to quantify HH-related joint damage in an objective, reproducible, and clinically feasible way.

Unlike conventional radiography, high-resolution peripheral quantitative computed tomography (HR-pQCT) allows for a three-dimensional, in vivo visualization and semi-automated assessment of joint space morphology and bone microarchitecture at spatial resolutions in the order of 100 μm [24]. Using this imaging modality, a recent study by Jandl et al. was able to identify altered bone microarchitecture of the distal tibia in patients with HH [25]. In addition, first cross-sectional clinical studies have successfully applied HR-pQCT-based joint space analysis on rheumatoid arthritis patients and were able to identify significant changes in joint space morphology at the MCPs [26, 27]. However, to date, these techniques have not been used to evaluate MCP joint space morphology in HH patients despite the growing need for reliable radiological HH joint outcome measures with several novel iron overload targeting therapeutics on the horizon [28] [29] and in clinical trial phase (NCT04059406, NCT04364269).

In addition, it is unclear, if and how the automated HR-pQCT-based joint space quantification algorithm would perform, given that HH arthropathy is known for its very narrow joint spaces. Thus, the aims of this exploratory study were (i) to determine the feasibility and performance of the HR-pQCT-based joint space width (JSW) algorithm in the MCP joints of HH patients, (ii) to quantify MCP joint morphology in HH patients, and (iii) to investigate the relationship between HR-pQCT-derived morphological joint parameters and clinical parameters in HH patients. Furthermore, we were interested to determine in an additional subanalysis potential differences in MCP joint space morphology between HH patients and age-matched healthy controls.

## Materials and Methods

### HH Patient Characteristics, Physical Exam, and History Taking

Twenty-four men and women with HH were enrolled in this study. HH patients were seen in the outpatient clinic at the Department of Rheumatology, University of Erlangen, Germany, as part of their routine clinical follow-up visit. Only patients with known C282Y homozygous HH were included in the study, whereas patients with other bone affecting conditions such as hyperthyroidism, hyperparathyroidism, chronic renal disease, or rheumatologic diseases were excluded. Additional exclusion criteria encompassed pregnant or breast-feeding women. All patients underwent a physical exam in which weight and height were recorded and in which pain levels at both hands and both wrists were assessed on a visual analogue scale from 1 to 10. In addition, a detailed history was taken by the same rheumatologist (SF) as part of which duration of HH, the presence or absence of treatment, the total number of phlebotomies since HH diagnosis, and the annual number of phlebotomies were recorded. All subjects gave written informed consent prior to enrollment. The study was HIPAA compliant and approved by the Institutional Review Board (IRB) of the University Clinic of Erlangen.

### HR-pQCT Imaging and Image Analysis in HH Patients

All HH patients underwent high-resolution peripheral quantitative computed tomography (HR-pQCT) scanning at the clinical dominant hand on the same clinical HR-pQCT system (XtremeCT I, Scanco Medical AG, Brüttisellen, Switzerland). In order to reduce motion artifacts during acquisition, the forearm was immobilized in a carbon fiber cast prior to the scan. A single dorsal-palmar scout radiograph of the hand was next acquired, and a reference line was placed at the apex of the distal metacarpal head of MCP 3 such that the encompassed tomographic scan region covered 13.53 mm in distal and proximal directions. In each patient, a stack of 330 images was acquired covering the metacarpophalangeal joints MCP 2, 3, and 4 and using a scan protocol as described before [26]. Images were reconstructed to a 1536 × 1536 matrix, the field of view spanned 12.6 cm, allowing for a final isotropic voxel size of 82 μm and a true spatial resolution of about 130 μm [30]. For each MCP, image quality including the presence and severity of motion artifacts was scored using a grading scheme established initially by Pialat et al. for grading of HR-pQCT images of the distal radius and tibia [31]. MCP joints with image quality scores of 4 or 5 were excluded from further analysis as were MCP joints whose entire joint surfaces were not fully contained in the 330-slice stack. In total, 69 out of 72 MCP joints fulfilled the image quality criteria and were used for further analysis.

**Fig. 1:**
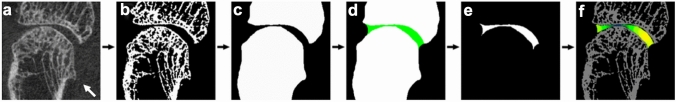
HR-pQCT image processing pipeline as published previously [26] and as now applied to the MCP 2 joint of a 55-year-old man who was diagnosed with HH at age 47. **a** Original coronal HR-pQCT image of the MCP 2 joint. Note the beak-like osteophyte at the metacarpal head (arrow). Figure 1 **b**, binarized image. After semi-automated contouring of the metacarpal bone and the adjacent proximal phalanx, the mineralized bone structure of both bones was automatically segmented from the grayscale image **c** Periosteal surface bone masks and the joint space mask **d **(in green) and **e** (in white) were then generated using several morphological image processing techniques. The joint space mask was then used to calculate all joint space related morphological parameters **f** Note the locally reduced joint space width as color-coded by dark green

In order to quantify MCP joint space morphology in the HH patients, we employed the 3D MCP joint space algorithm (also referred to as “UCSF MCP JSW algorithm”) to each MCP joint as previously described by Dr. Burghardt [26]. Details of the algorithm are summarized in Fig. 1. In brief, each MCP joint was first individually identified by semi-automatically drawing a simple circular contour around its distal metacarpal bone and its proximal phalanx. Adjacent sesamoid bones were excluded from the contours. After identification of the bones, the mineralized bone structure was automatically segmented from the grayscale image data and periosteal surface bone masks and the joint space mask were generated using morphological image processing as detailed previously [26]. Segmentation was considered successful if the automatic segmentation result of MCP joint contours was in agreement with the qualitative visual contour check performed by a radiology-trained physician with 5 years of experience in joint space segmentation (UH). From the joint space mask, the following 6 standard joint space morphometric parameters were calculated for each MCP joint: joint space volume (JSV), mean joint space width (JSW), JSW heterogeneity (JSW.SD), minimal and maximal JSW (JSW.MIN, JSW.MAX), and joint space width asymmetry (JSW.AS = JSW.MAX/JSW.MIN). This processing and quantification were performed individually for each MCP joint.

### Recruitment, HR-pQCT Imaging, and MCP Image Analysis in Healthy Controls

Details on the recruitment and HR-pQCT image acquisition of healthy controls and on the MCP joint space algorithm utilized for the subanalysis between HH patients and controls are provided in the Supplementary material, Section B.

### Statistical Analysis

Normal distribution of data was checked visually via Q-Q-plots and mathematically using Shapiro–Wilk testing. For normally distributed data, intergroup differences were assessed for each MCP joint indivdiually via independent *t*-tests or Pearson’s chi-squared tests, as appropriate. For not normally distributed data, Mann–Whitney-*U*-tests were carried out individually by MCP joint to determine intergroup differences. Correlations between HR-pQCT microstructural joint parameters and clinical outcomes were assessed using Spearman´s correlations. Spearman´s Rho (*ρ*) (correlation coefficients) were reported. In order to compare differences in MCP joint space morphology between HH patients and controls, a subanalysis was performed for which HH patients were 1:1 matched to healthy controls by sex and age, using 5 years of age strata. *p*-values below 0.05 were considered statistically significant. All statistical analyses were carried out using SPSS version 27 (IBM, Armonk, NY, USA).

## Results

### Characteristics of HH Patients and Controls

Subject characteristics of all 24 HH patients are presented in Table 1, for 1:1 matched controls in Tables 3 and 4. HH patients were mostly male (about 67%), with a mean age of 54.7 ± 10.8 years and had been diagnosed with HH approximately 10.2 ± 9.2 years ago. 19 out of the 24 HH patients had received regular treatment by phlebotomies, 2 patients had been without treatment and for three HH patients, information on HH-related treatment was not available. On average, HH patients had been treated with about 6 phlebotomies annually and had received approximately 60.6 ± 66.0 phlebotomies since HH diagnosis. With respect to arthralgias, we found that about 71% of all HH patients reported joint pain in at least one hand at the time of the study visit. When looking at sex differences, we noticed that HH men had received numerically more phlebotomies than HH women since diagnosis (*p* = 0.085). However, all other anthropometrics were comparable between sexes including the proportion of HH individuals with hand arthralgias per group and the time since HH diagnosis.

**Table 1:** Patient characteristics given for all 24 study participants with HH and stratified by gender

	Means ± SD			*p* value
	All HH patients (*n* = 24)	HH men (*n* = 16)	HH women (*n* = 8)	HH men vs. HH women
Demographics				
Age [years]	54.7 ± 10.8	53.1 ± 11.9	58.0 ± 7.8	0.301
BMI [kg/m^2^]	26.6 ± 4.5	27.3 ± 4.7	25.1 ± 3.9	0.245
Height (cm)	175.5 ± 11.2	180.1 ± 9.7	165.4 ± 6.8	**0.002**
Gender, female *n* [%]	8 [33.3]	0 [0%]	8 [100%]	n.a
Time since HH diagnosis [years]	10.2 ± 9.2	10.8 ± 11.1	9.0 ± 3.9	0.497
Phlebotomies since diagnosis *n*	60.6 ± 66.0	80.8 ± 74.5	28.7 ± 33.9	*0.085*
Annual rate of phlebotomies *n*	6.6 ± 11.1	7.9 ± 13.4	4.2 ± 4.8	0.563
Arthralgias in at least one hand *n* [%]	17 [70.8]	10 [62.5]	7 [87.5]	0.168
MCP 2				
JSV [mm^3^]	131.83 ± 32.13	141.39 ± 33.64	112.73 ± 18.48	**0.043**
JSW [mm]	1.65 ± 0.34	1.71 ± 0.39	1.53 ± 0.15	0.134
JSW.MIN [mm]	0.67 ± 0.47	0.60 ± 0.49	0.80 ± 0.43	0.305
JSW.MAX [mm]	2.18 ± 0.30	2.24 ± 0.33	2.05 ± 0.15	*0.062*
JSW.AS	9.17 ± 10.35	10.95 ± 11.03	5.61 ± 8.34	0.133
JSW.SD [mm]	0.33 ± 0.10	0.35 ± 0.10	0.29 ± 0.08	0.134
MCP 3				
JSV [mm^3^]	124.51 ± 37.46	137.44 ± 40.13	101.89 ± 17.35	**0.024**
JSW [mm]	1.46 ± 0.25	1.49 ± 0.30	1.40 ± 0.15	0.344
JSW.MIN [mm]	0.63 ± 0.42	0.62 ± 0.46	0.66 ± 0.38	0.856
JSW.MAX [mm]	2.08 ± 0.28	2.18 ± 0.28	1.90 ± 0.15	**0.011**
JSW.AS	7.77 ± 8.39	7.95 ± 8.00	7.47 ± 9.59	0.539
JSW.SD [mm]	0.33 ± 0.08	0.36 ± 0.08	0.29 ± 0.06	*0.058*
MCP 4				
JSV [mm^3^]	112.08 ± 23.24	124.88 ± 16.54	88.08 ± 11.84	**< 0.001**
JSW [mm]	1.42 ± 0.20	1.48 ± 0.21	1.31 ± 0.13	**0.043**
JSW.MIN [mm]	0.75 ± 0.34	0.78 ± 0.40	0.69 ± 0.20	0.195
JSW.MAX [mm]	1.94 ± 0.22	2.03 ± 0.18	1.77 ± 0.21	**0.006**
JSW.AS	4.67 ± 6.47	5.66 ± 7.89	2.82 ± 1.05	0.651
JSW.SD [mm]	0.29 ± 0.09	0.31 ± 0.11	0.26 ± 0.06	0.243

Shown are unadjusted means with standard deviation (SD). Intergroup differences were calculated using Mann–Whitney-*U*-tests or independent *t*-tests as appropriate

Significant *p*-values (*p* < 0.05) are marked in bold print, statistical trends are printed in *italics*

### Segmentation Performance and Algorithm Completion Rates of Joint Space Analysis in HH Patients

**Fig. 2:**
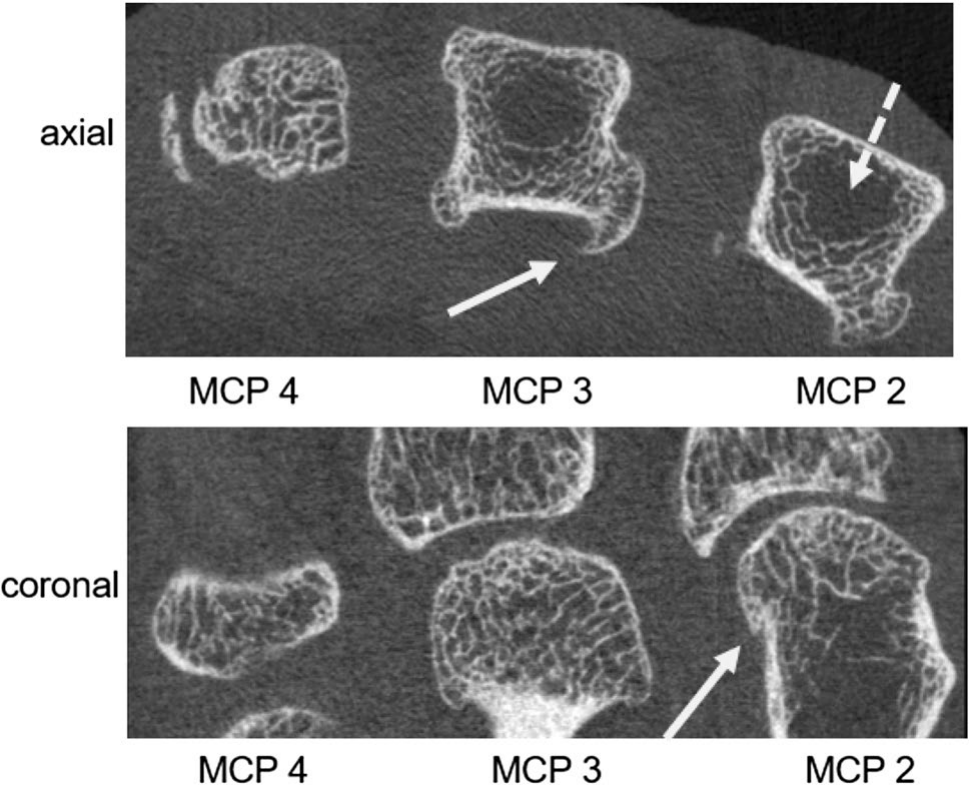
Axial and coronal HR-pQCT images showing metacarpal heads 2–4 (axial view) and MCP joints 2–4 (coronal view) of a patient with HH. Note the distinct features of hemochromatosis arthropathy: characteristic hook- or beak-like osteophytes at the metacarpal heads are seen (white arrows) along with small cysts. In addition, areas of sparse to absent trabecular bone structure are noted in the center of the metacarpal heads 2 and 3 (dashed white arrow) indicative of a HH-related impairment of bone microarchitecture

**Fig. 3:**
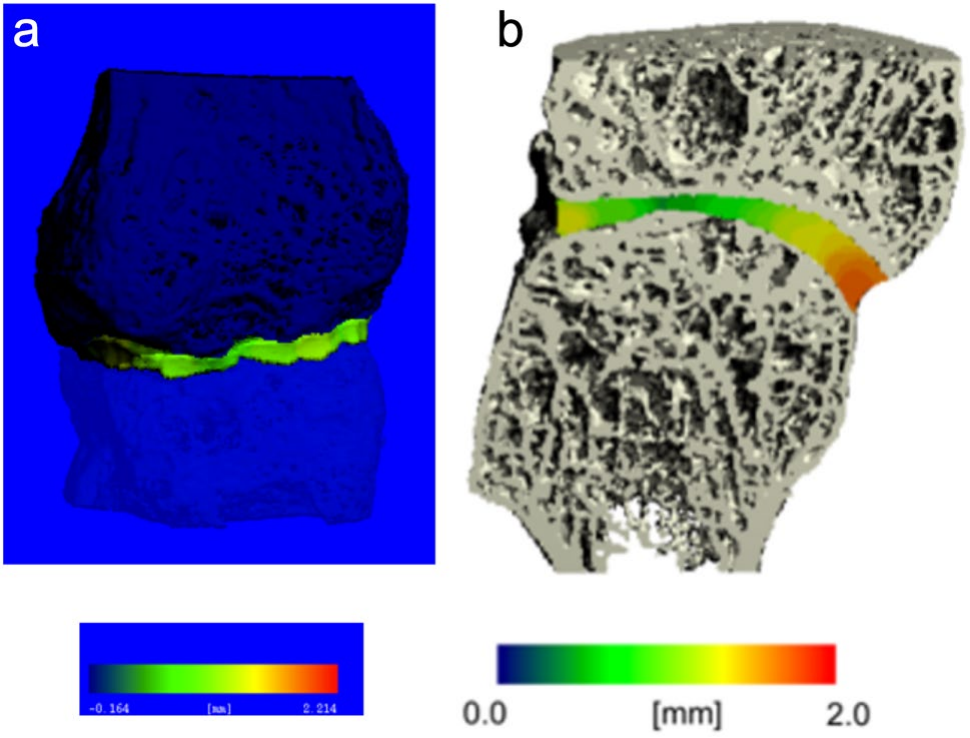
**a** Initial 3D rendering of the segmentation result of the MCP 4 joint of a 72-years-old male, diagnosed with HH 31 years ago. The metacarpal head is depicted in light blue, while the base of the proximal phalanx is shown in dark blue. The local joint space width was mapped in the joint space in pseudo-color. Blue and green colors reflect a narrow joint space width, while red colors code for a broader joint space width. Note the very narrow and irregular joint space depicted in light green. **b** Final 3D surface reconstruction of the MCP 3 joint of a 55-years-old male (BMI 21.9 kg/m^2^) diagnosed with HH 8 years ago. The local joint space width was mapped in the joint space in pseudo-color. Blue and green colors reflect a narrow joint space width, while red colors signify a broader joint space width

From the 24 HH patients, a total of 72 MCP joints (24 MCP 2, 24 MCP 3, 24 MCP 4 joints) were available to be processed via the HR-pQCT-based UCSF JSW algorithm [26]. Representative axial cross-sectional HR-pQCT images of a HH patient are shown in Fig. 2 and representative 3D visualizations of the joint space segmentation and morphometric results are presented in Fig. 3. Due to poor image quality related to motion, two out of 72 MCP joints were excluded from the analysis, one MCP joint was excluded due to ankylosis of the joint space. For the remaining 69 MCP joints, the joint spaces were successfully segmented automatically for 55 cases at the first attempt (79.7% overall; 75% of MCP 2 joints, 81.8% of MCP 3 joints, 82.6% of MCP 4 joints). Additional semi-manual segmentation intervention to separate the individual bones was required for 14 cases (20.3%). This correction step involved a short (< 5 min) semi-automatic coarse contour of the metacarpal head by the operator to differentiate it from the proximal phalangeal base.

When looking at the minimum joint space width (JSW.MIN) needed, above which the JSW software would be able to correctly segment the MCP joints at a success rate of 100% at the first attempt, we found that for all MCP joints, the minimum mean JSW value was 0.082 mm (i.e., one voxel wide). When we evaluated the segmentation performance of the MCP JSW quantification software stratified by sex, we observed that in HH men and HH women similar proportions of MCP joints were successfully automatically segmented by the software (87.5% of all 24 female MCP joints vs. 75.6% of all 45 male MCP joints, *p* = 0.245). Only 12.5% of MCP joints from female HH patients and 24.4% from male HH patients required additional semi-manual correction (*p* = 0.245).

### Joint Space Parameters Measured by High-Resolution Peripheral Quantitative Computed Tomography (HR-pQCT) in HH Men and Women

With respect to MCP joint morphology, we observed that the joint space volume (JSV) was significantly larger in HH men compared to HH women in all three MCP joints (+ 25.4% at MCP 2, + 34.9% at MCP 3, + 41.8% at MCP 4, 0.001 < *p* < 0.043). Additionally, mean JSW at the MCP 4 was significantly larger (+ 13%, *p* = 0.043), and the maximal JSW was significantly higher (up to + 14.7%) in the MCP 3 and 4 (*p* < 0.011) in HH men than HH women. Also, a strong trend (+ 9.3% JSW.MAX, *p* = 0.062) toward a larger maximal JSW was observed in the MCP 2 joint in HH men compared to HH women. All other MCP structural and morphological parameters were comparable between HH men and HH women.

### Correlations of MCP Joint Morphological Parameters and Clinical Measures in HH patients

With respect to clinical correlations (Table 2), we found that in the overall cohort, time since HH diagnosis was significantly correlated with MCP 4 JSW asymmetry (JSW.AS: *ρ* = 0.463, *p* = 0.040) and MCP 4 JSW heterogeneity (JSW.SD: *ρ* = 0.499, *p* = 0.025). In addition, the total number of phlebotomies since diagnosis was moderately correlated with the JSW.SD at all MCP sites, reaching statistical significance at MCP 3 and 4 (0.492 < *ρ* < 0.535, *p* < 0.045) and approaching significance at MCP 2 (*ρ* = 0.432, *p* = 0.073). At MCP 2, there were also statistical trends for correlation between JSW asymmetry and the number of phlebotomies since HH diagnosis (*ρ* = 0.460, *p* = 0.055). Estimated therapy intensity as indicated by the yearly rate of phlebotomies was for the overall cohort not significantly correlated with any of the joint structural parameters (data not shown).

**Table 2:** Spearman´s rho correlations showing the associations between HR-pQCT-derived joint space measures obtained from MCP joints 2, 3, and 4 with clinical parameters in HH men and HH women

	Time since HH Diagnosis [years]			Number of phlebotomies since HH diagnosis		
	All HH patients (*n* = 24)	HH men (*n* = 16)	HH women (*n* = 8)	All HH patients (*n* = 24)	HH men (*n* = 16)	HH women (*n* = 8)
MCP 2						
JSV [mm^3^]	− 0.226	− 0.241	0.074	− 0.104	− 0.683**	0.487
JSW [mm]	− 0.337	− 0.372	− 0.148	− 0.139	− 0.519	0.216
JSW.MIN [mm]	− 0.198	− 0.406	0.224	− 0.419*	− 0.668**	0.109
JSW.MAX [mm]	− 0.305	− 0.331	− 0.136	− 0.100	− 0.575*	0.255
JSW.AS	0.128	0.388	− 0.148	0.460*	0.589*	0.072
JSW.SD [mm]	0.226	0.507*	− 0.185	0.432*	0.515	0.126
MCP 3						
JSV [mm^3^]	− 0.035	− 0.021	0.704*	− 0.003	− 0.594*	0.541
JSW [mm]	− 0.333	− 0.442	0.185	− 0.199	− 0.611*	0.054
JSW.MIN [mm]	− 0.273	− 0.419	0.264	− 0.146	− 0.536	0.413
JSW.MAX [mm]	− 0.251	− 0.208	0.000	0.188	− 0.409	0.342
JSW.AS	0.238	0.558*	− 0.206	0.207	0.669**	− 0.336
JSW.SD [mm]	0.150	0.481	− 0.371	0.535**	0.703**	0.180
MCP 4						
JSV [mm^3^]	− 0.184	− 0.302	0.630	0.333	− 0.402	0.577
JSW [mm]	− 0.043	− 0.050	0.037	0.097	− 0.170	− 0.324
JSW.MIN [mm]	− 0.317	− 0.325	− 0.229	− 0.043	− 0.381	0.435
JSW.MAX [mm]	0.243	0.220	0.617	0.353	− 0.062	0.236
JSW.AS	0.463**	0.416	0.617	0.184	0.314	− 0.145
JSW.SD [mm]	0.499**	0.443	0.704*	0.492**	0.274	0.505

Data are presented for all HH patients (*n* = 24) and stratified by gender. Reported are Spearman´s Rho (*ρ*) correlation coefficients

Significant correlations (*p* < 0.05) are marked in bold and are marked by two asterisks. Correlations with statistical trends of significance are printed in bold italics are marked by one asterisk

In HH men, time since HH diagnosis showed a trend for moderate correlation with JSW heterogeneity at MCP 2 and JSW asymmetry at MCP 3 (0.507 < *ρ* < 0.558, *p* < 0.064). With respect to the total number of phlebotomies since HH diagnosis, significant correlations were seen in HH men with joint space volume and minimal JSW at MCP 2 (0.668 < *ρ* < 0.683, 0.020 < *p* < 0.025) and with JSW asymmetry and JSW heterogeneity at MCP 3 (0.669 < *ρ* < 0.703, 0.035 < *p* < 0.049).

In HH women, no significant correlation between time since HH diagnosis or the number of phlebotomies and MCP joint morphological parameters was found. Estimated therapy intensity as defined by the annual rate of phlebotomies did not significantly correlate with the HR-pQCT-derived MCP joint space parameters (data not shown).

### Joint Space Morphology Parameters in HH Patients with and Without Joint Pain

In an exploratory subanalysis, we investigated the differences in MCP joint morphology between HH patients with and without hand arthralgia (see Supplementary Table 1). We found that HH patients with hand arthralgia had a higher mean age (56.1 ± 10.3 years vs. 45.0 ± 15.4 years) and a longer HH duration (11.9 ± 10.2 vs. 5.0 ± 2.0 years) compared to HH patients without pain; however, these differences were not large enough to translate into statistical significances. HH patients with joint pain also showed lower joint space volume (JSV) at all three MCP joints, reaching statistical significance at MCP 2 and MCP 4 (MCP 2: *p* = 0.009; MCP 4: *p* = 0.048). Additionally, maximal joint space width (JSW.MAX) was significantly smaller at MCP 2 (*p* = 0.040) in HH patients with hand arthralgia compared to HH patients without arthralgia.

### Differences in MCP Joint Space Morphology Between HH Patients and Controls

In order to better understand the differences in MCP joint morphology between HH patients and healthy controls, we performed additional exploratory subanalyses for which HH patients were matched 1:1 by sex and age to their respective healthy controls. Results of these subanalyses are shown in Tables 3 and 4. HH women exhibited at the MCP 3 joint a significant, 1.8 times larger JSW asymmetry and an about 1/3 larger JSW heterogeneity relative to age-matched healthy control women (MCP 3 JSW.AS: + 180% *p* = 0.025; JSW.SD: + 37.5%, *p* = 0.026). Minimum JSW was significantly smaller in HH women compared to controls (MCP 3: JSW.MIN: − 41.8%, *p* = 0.022) with mean JSW showing also a statistical trend toward being smaller in HH women relative to controls (MCP 3: JSW: − 13.9%, *p* = 0.086). Similar to the MCP 3 joint, we observed at the MCP 2 joint numerically larger JSW asymmetry and larger JSW heterogeneity and numerically smaller minimal, mean, and maximal JSW in HH women. However, these results did not reach statistical significance with the exception of mean JSW, where a statistical trend toward a smaller mean JSW was noted in HH women (MCP 2: JSW: − 13.3%, *p* = 0.087).

**Table 3:** Results from the exploratory subanalysis comparing MCP joint space morphology between HH women and age-matched healthy control women

	Means ± SD		*p* value HH women vs. control women
	HH women (*n* = 8)	Healthy control women—age matched (*n* = 8)	
Demographics			
Age [years]	58.0 ± 7.8	58.0 ± 8.2	0.975
BMI [kg/m^2^]	25.1 ± 3.9	25.4 ± 3.0	0.393
Height (cm)	165.4 ± 6.8	165.9 ± 6.5	0.950
Gender, female n [%]	8 [100%]	8 [100%]	1.000
Time since HH diagnosis [years]	9.0 ± 3.9	n.a.	n.a.
Phlebotomies since diagnosis *n*	28.7 ± 33.9	n.a.	n.a.
Annual rate of phlebotomies *n*	4.2 ± 4.8	n.a.	n.a.
Arthralgias in at least one hand *n* [%]	7 [87.5]	n.a.	n.a.
HR-pQT-derived MCP joint space parameters			
MCP 2			
JSV [mm^3^]	81.52 ± 14.90	87.5 ± 11.6	0.208
JSW [mm]	1.56 ± 0.16	1.80 ± 0.26	*0.087*
JSW.MIN [mm]	0.83 ± 0.41	1.27 ± 0.36	0.140
JSW.MAX [mm]	2.64 ± 0.2	2.78 ± 0.12	0.115
JSW.AS	5.06 ± 5.23	2.39 ± 0.83	0.208
JSW.SD [mm]	0.33 ± 0.06	0.28 ± 0.07	0.270
MCP 3			
JSV [mm^3^]	79.0 ± 7.77	87.8 ± 17.10	0.185
JSW [mm]	1.43 ± 0.16	1.66 ± 0.26	*0.086*
JSW.MIN [mm]	0.71 ± 0.36	1.22 ± 0.21	**0.022**
JSW.MAX [mm]	2.58 ± 0.31	2.65 ± 0.25	0.327
JSW.AS	6.21 ± 6.07	2.21 ± 0.31	**0.025**
JSW.SD [mm]	0.33 ± 0.06	0.24 ± 0.07	**0.026**

Patient characteristics and HR-pQCT-derived joint space parameters of metacarpophalangeal joints (MCP) 2 and 3 are presented for women suffering from HH (*n* = 8) and for healthy control women (*n* = 8). Healthy control women were matched 1: 1 to HH women by age, using 5 years age strata. Intergroup differences were assessed using paired *t*-test, if data were normally distributed, or Wilcoxon-signed-rank test, if data were not normally distributed

*HR-pQCT* High-resolution peripheral quantitative computed tomography,  *n.a.* not applicable

Significant *p*-values (*p* < 0.05) are marked in bold print, statistical trends are printed in italics*

^*^For comparability reasons, all above-mentioned MCP HR-pQCT data were analyzed using the SPECTRA consensus MCP joint space algorithm [44] as described in detail in the Supplementary material

**Table 4:** Results from the exploratory subanalysis comparing MCP joint space morphology between HH men and age-matched healthy control men

	Means ± SD		*p* value HH men vs. control men
	HH men (*n* = 5)	Healthy control men—age matched (*n* = 5)	
Demographics			
Age [years]	59.8 ± 12.5	60.8 ± 15.7	0.639
BMI [kg/m^2^]	29.9 ± 6.1	28.0 ± 3.9	0.572
Height (cm)	175.8 ± 8.2	173.6 ± 10.5	0.587
Gender, female n [%]	8 [100%]	8 [100%]	1.000
Time since HH diagnosis [years]	19.6 ± 14.9	n.a.	n.a.
Phlebotomies since diagnosis *n*	95.0 ± 83.8	n.a.	n.a.
Annual rate of phlebotomies *n*	5.0 ± 1.4	n.a.	n.a.
Arthralgias in at least one hand *n* [%]	4 [80.0]	n.a.	n.a.
HR-pQT-derived MCP joint space parameters			
MCP 2			
JSV [mm^3^]	106.33 ± 33.60	102.52 ± 21.81	0.864
JSW [mm]	1.56 ± 0.37	1.79 ± 0.04	0.239
JSW.MIN [mm]	0.31 ± 0.33	1.00 ± 0.34	**0.043**
JSW.MAX [mm]	2.77 ± 0.20	2.83 ± 0.10	0.654
JSW.AS	14.2 ± 6.37	3.28 ± 1.66	**0.043**
JSW.SD [mm]	0.46 ± 0.14	0.37 ± 0.12	0.208
MCP 3			
JSV [mm^3^]	92.03 ± 29.36	117.85 ± 13.74	0.200
JSW [mm]	1.45 ± 0.28	1.72 ± 0.26	0.234
JSW.MIN [mm]	0.34 ± 0.36	1.08 ± 0.36	**0.043**
JSW.MAX [mm]	2.76 ± 0.24	2.76 ± 0.11	0.997
JSW.AS	12.75 ± 6.28	2.78 ± 0.92	**0.020**
JSW.SD [mm]	0.49 ± 0.14	0.34 ± 0.10	0.144

Patient characteristics and HR-pQCT-derived joint space parameters of metacarpophalangeal joints (MCP) 2 and 3 shown for men suffering from HH (*n* = 5) and for healthy control men (*n* = 5). Healthy control men were 1: 1 matched to HH men by age, using 5 years age strata. Intergroup differences were assessed using paired *t*-test, if data were normally distributed, or Wilcoxon-signed-rank test, if data were not normally distributed

*HR-pQCT* high-resolution peripheral quantitative computed tomography,  *n.a.* not applicable

Significant *p*-values (*p* < 0.05) are marked in bold print*

^*^For comparability reasons, all above-mentioned MCP data were analyzed using the SPECTRA consensus MCP joint space algorithm [44] as described in detail in the Supplementary material

For HH men versus controls, a similar, but more pronounced and uniform pattern of MCP joint space differences was noted consistently throughout MCP 2 and 3 joints. At both MCP 2 and 3 joints, JSW asymmetry was significantly and around 3 × times larger in HH men relative to healthy men (MCP 2: JSW.AS: + 323%, *p* = 0.043; MCP 3: JSW.AS: + 359%, *p* = 0.020), while minimum JSW was at both sites around 2 × smaller (MCP 2: JSW.MIN:− 225%, *p* = 0.043; MCP 3: JSW.AS: − 216%, *p* = 0.043). All other joint space parameters did not differ between HH men and their age-matched controls.

## Discussion

In this exploratory study, we investigated MCP joints of men and women with HH via HR-pQCT. Although HR-pQCT is mostly used to quantify bone microarchitecture of the distal radius/tibia in the context of fracture risk assessment [32], we focused in this study on quantification of MCP joints, as particularly MCP 2 and MCP 3 are most commonly affected in HH [33] and can therefore serve as sentinels for HH joint involvement. An automated 3D joint space quantification technique—the UCSF MCP JSW algorithm—previously validated for measuring MCP joint space morphology in rheumatoid arthritis (RA) patients [26], was applied to the HR-pQCT images of HH patients. At the time of the study, it was unclear if and how this algorithm would perform on arthropathies with very narrow joint spaces such as HH. Therefore, investigating the feasibility and performance of the HR-pQCT-derived JSW quantification algorithm in MCP joints of HH patients was one of the main aims of this study.

Joint space width quantification utilizing the HR-pQCT-derived joint space quantification algorithm was feasible in the MCP joints of HH patients. Unlike in a RA study by Burghardt et al. which used the same HR-pQCT-derived JSW algorithm on MCPs of RA patients, and in which about 20% of MCP images had no suitable image quality and had to be excluded due to severe motion, only about 2.8% of MCPs of our HH patient study had to be excluded due to severe motion artifacts. This low frequency of motion artifacts observed in our HH patients emphasizes the fact that, from a clinical perspective, HH patients are well suited for MCP joint space evaluation via HR-pQCT. In both sexes and in the overall HH cohort, about 80% of MCP joints were segmented successfully by the automated MCP quantification algorithm at first attempt despite relatively narrow joint space patterns. In approximately 20% or less of MCP HH cases, the segmentation required additional manual intervention to differentiate the metacarpal head and proximal phalangeal cup due to direct bony contact resulting in a localized complete loss of intraarticular joint space. This additional manual segmentation step required only a short (ca 5 min) semi-automatic circling of the metacarpal head by the operator. For rheumatoid arthritis, a slightly higher success rate has been reported using the same software algorithm [26]. However, this latter study excluded a higher percentage of MCP joints due to motion artifacts and it remains unclear how including those MCP joints may have affected the segmentation success rates [26]. All other studies using similar JSW algorithms did not give information on MCP segmentation success rates nor commented on the necessity of additional operator intervention needed to achieve successful MCP bone and joint space segmentation [27, 34]. Given that only one in five MCP joints of our HH patients required a manual segmentation, and that the algorithm only failed in HH MCPs with a minimum JSW of 0.082 mm or less (at or below voxel size), we feel confident that use of this JSW algorithm should be feasible for future clinical HR-pQCT-based HH studies of MCP joint morphology and should only require a small, manageable amount of operator intervention.

Another important finding of this HH study was that it analyzed joint space morphology for the overall HH cohort and separated by sex. We found that compared to women suffering from HH, HH men consistently showed significantly larger joint space volumes at all MCP joints and a significantly larger maximal JSW at MCP 3 and 4. At first glance, these findings seem to contrast the recent bone microstructural results obtained via HR-pQCT by Jandl et al. in a small group of 10 HH patients [25] who did not observe any bone microstructural differences between HH men and women at the distal tibia and radius [25]. However, unlike our study, this study evaluated exclusively bone microarchitecture at peripheral long bones and did not include any joint analysis or any periarticular bone microstructural assessments making it only partially comparable. Given that an exploratory comparison of MCP joint morphology in healthy men and women (data shown in the Supplementary material) also demonstrated significantly larger JSV for healthy men at the MCP 3 level, the differences in JSV noted between HH men and women may indeed be partly influenced by sex and thus have to be interpreted with caution. Notably, and unlike for HH men versus HH women, other parameters such as JSW.MAX did not differ between healthy men and women (data shown in Supplementary material). This suggests that this parameter may be less affected by sex and could indicate that HH may affect certain parameters of MCP joint space morphology differently in HH men and HH women. However, given the small sample size of our exploratory study, larger validation studies are needed in order to investigate further sex-specific joint morphological differences in HH patients.

When comparing MCP joint morphology between HH patients and age- and sex- matched controls, our exploratory subanalysis revealed a larger joint space width asymmetry (JSW.AS) and a smaller minimal joint space width (JSW.MIN) at all MCP levels in HH patients, reaching significance for HH men at MCP 2 and 3 and for women at MCP 3. These results are in line with our initial observations of narrower and more asymmetric MCP joint spaces in this patient group when we visually inspected the HR-pQCT images for image quality. Moreover, our findings also confirm and extend on previous radiographic HH studies [18] [35], in which MCP joint space narrowing and MCP joint space irregularities due to erosions or bony enlargements (= osteophytes) of metacarpal heads were noted in HH patients by visual grading of hand radiographs. Particularly, our study findings are in good agreement with the results of the few advanced imaging studies available [33] [36] in which MCP joint space narrowing was detected via ultrasound or via low-field MRI of the hands in HH patients. However, in both latter studies, no healthy controls were included. Therefore, to the best of our knowledge, this is the first high-resolution study assessing exploratively and quantitatively MCP joint space morphology in HH compared to healthy controls. From a pathophysiological point of view, the larger JSW asymmetry and smaller JSW.MIN observed in MCP joints of HH patients may be the morphological consequence of an ongoing HH-induced joint destruction, in which cartilage degradation may have been accelerated compared to controls by the cumulative effects of iron toxicity [37], neutrophil invasion [38], and impaired BMP signaling [9]. However, future studies combining clinical imaging with histology are needed to validate our findings in a larger HH cohort and to shed more light on the underlying pathomechanisms of joint degradation in HH.

Another relevant finding of our study was that we observed moderate correlations between MCP joint space parameters and clinical features of HH. Especially joint space asymmetry (JSW.AS) and heterogeneity (JSW.SD) were correlated with disease duration, particularly in HH men. Interestingly, the number of phlebotomies was also significantly and positively correlated with joint space asymmetry (JSW.AS) and heterogeneity (JSW.SD) in the overall cohort and in HH men, predominantly at the MCP 3 and 4 joint. Given that number of phlebotomies and disease duration are significantly correlated, this finding could also be interpreted such that the increasing joint irregularities are more driven by the HH disease duration than by the treatment. On the other hand, therapeutic phlebotomy may not be very effective in preventing HH-related cartilage destruction. This result is somewhat surprising as one might have thought that regular phlebotomies and a regular reduction of iron blood levels would help reduce iron load in the body and joints and therefore help preserve joint space morphology. However, in line with our findings, prior studies have reported that regular removal of excess blood via phlebotomies does not stop or improve HH-related joint alterations [39, 40]. Other mechanisms such as a localized cellular iron dysregulation in chondrocytes in the HH joint spaces may also occur [41], triggering a cascade of inflammatory, oxidative stress and epigenetic responses which may perpetuate HH-related joint disease, even in the presence of blood iron levels getting regularly normalized through phlebotomies [42]. Along these lines, a recent miRNA study showed a significant upregulation of several serum miRNAs involved in the posttranscriptional iron metabolism (e.g., miR-141, miR-182, miR-31) in HH patients, of which the latter (miR-31) serves as an important regulator of osteoclast-associated bone resorption [43]. Relation between disease duration and number of phlebotomies was only found in men but not in women. If this finding may be based on the smaller sample size in women or due to the fact that women loose blood and iron through their menstruation remains open at this point. Future longitudinal studies are needed to compare long-term joint alterations in HH men and women in order to elucidate if these changes progress similarly in HH men and women.

Our study has several limitations. First, with 24 subjects, the sample size of our study is small. However, despite the small sample size, we yielded significant results indicating that our study had enough statistical power. In addition, this study was of exploratory nature in order to test the feasibility of JSW measurement in HH using HR-pQCT and a special analysis algorithm. Given that our study showed that such an approach is feasible further studies in larger cohorts can follow. A second limitation of this study was the lack of clinical laboratory measures of iron homeostasis such as ferritin and of inflammation markers which would have allowed additional interesting correlations. Another limitation of this study was that no conventional hand radiographs were acquired at the time of HR-pQCT-based MCP joint scanning and no data on the overall skeletal status (e.g., distal radial bone microarchitecture measurements) were available which prevented us from performing additional clinical correlations.

In conclusion, our findings show that despite the very narrow joint spaces in HH joints, assessment of JSW parameters via HR-pQCT-based MCP joint space quantification is technically feasible, performs well, and requires relatively low operator intervention. In addition, our exploratory study findings indicate that HR-pQCT-based MCP joint space assessment was able to identify and quantify HH-related structural differences in MCP joint morphology relative to controls and thus define the microstructural joint burden of HH. However, further and larger studies are needed to validate our findings and to determine if HR-pQCT-based joint space quantification in MCP joints may qualify as outcome measure for future clinical HH trials.

## Supplementary Information

Below is the link to the electronic supplementary material.

Supplementary file1 (DOCX 41 KB)

## Data Availability

Data are available upon reasonable request.

## References

[1] Pietrangelo A (2009) Hereditary hemochromatosis—A new look at an old disease. In: 10.1056/NEJMra031573. 10.1056/NEJMra031573 15175440

[2] Feder JN, Gnirke A, Thomas W (1996). A novel MHC class I-like gene is mutated in patients with hereditary haemochromatosis. Nat Genet.

[3] Bridle KR, Frazer DM, Wilkins SJ (2003). Disrupted hepcidin regulation in HFE-associated haemochromatosis and the liver as a regulator of body iron homoeostasis. Lancet.

[4] Mattman A, Huntsman D, Lockitch G (2002). Transferrin receptor 2 (TfR2) and HFEmutational analysis in non-C282Y iron overload: identification of a novel TfR2 mutation. Blood.

[5] Montosi G, Donovan A, Totaro A (2001). Autosomal-dominant hemochromatosis is associated with a mutation in the ferroportin (SLC11A3) gene. J Clin Invest.

[6] Niederau C, Fischer R, Purschel A (1996). Long-term survival in patients with hereditary hemochromatosis. Gastroenterology.

[7] von Kempis J (2001). Arthropathy in hereditary hemochromatosis. Curr Opin Rheumatol.

[8] Adams PC, Speechley M (1996). The effect of arthritis on the quality of life in hereditary hemochromatosis. J Rheumatol.

[9] Kiely PD (2018). Haemochromatosis arthropathy—a conundrum of the Celtic curse. J R Coll Physicians Edinb.

[10] Richardson A, Prideaux A, Kiely P (2017). Haemochromatosis: unexplained metacarpophalangeal or ankle arthropathy should prompt diagnostic tests: findings from two UK observational cohort studies. Scand J Rheumatol.

[11] Kowdley KV, Brown KE, Ahn J, Sundaram V (2019). ACG clinical guideline: hereditary hemochromatosis. Am J Gastroenterol.

[12] Radford-Smith DE, Powell EE, Powell LW (2018). Haemochromatosis: a clinical update for the practising physician. Intern Med J.

[13] Carroll GJ, Breidahl WH, Bulsara MK, Olynyk JK (2011). Hereditary hemochromatosis is characterized by a clinically definable arthropathy that correlates with iron load. Arthritis Rheum.

[14] Adams PC, Kertesz AE, Valberg LS (1991). Clinical presentation of hemochromatosis: a changing scene. Am J Med.

[15] Edwards CQ, Cartwright GE, Skolnick MH, Amos DB (1980). Homozygosity for hemochromatosis: clinical manifestations. Ann Intern Med.

[16] Nguyen C-D, Morel V, Pierache A (2020). Bone and joint complications in patients with hereditary hemochromatosis: a cross-sectional study of 93 patients. Ther Adv Musculoskelet Dis.

[17] Sinigaglia L, Fargion S, Fracanzani AL (1997). Bone and joint involvement in genetic hemochromatosis: role of cirrhosis and iron overload. J Rheumatol.

[18] Dallos T, Sahinbegovic E, Stamm T (2013). Idiopathic hand osteoarthritis vs haemochromatosis arthropathy–a clinical, functional and radiographic study. Rheumatology (Oxford).

[19] Sahinbegovic E, Dallos T, Aigner E (2010). Musculoskeletal disease burden of hereditary hemochromatosis. Arthritis Rheum.

[20] Kölmel S, Nowak A, Krayenbuehl P-A (2020). Iron overload associated symptoms and laboratory changes in the Swiss haemochromatosis cohort—when a clinician should become attentive. Swiss Med Wkly.

[21] Brissot P, Pietrangelo A, Adams PC (2018). Haemochromatosis. Nat Rev Dis Primers.

[22] Dallos T, Sahinbegovic E, Aigner E (2010). Validation of a radiographic scoring system for haemochromatosis arthropathy. Ann Rheum Dis.

[23] Peloschek P, Langs G, Weber M (2007). An automatic model-based system for joint space measurements on hand radiographs: initial experience. Radiology.

[24] Klose-Jensen R, Tse JJ, Keller KK (2020). High-resolution peripheral quantitative computed tomography for bone evaluation in inflammatory rheumatic disease. Front Med (Lausanne).

[25] Jandl NM, Rolvien T, Schmidt T (2020). Impaired bone microarchitecture in patients with hereditary hemochromatosis and skeletal complications. Calcif Tissue Int.

[26] Burghardt AJ, Lee CH, Kuo D (2013). Quantitative in vivo HR-pQCT imaging of 3D wrist and metacarpophalangeal joint space width in rheumatoid arthritis. Ann Biomed Eng.

[27] Barnabe C, Szabo E, Martin L (2013). Quantification of small joint space width, periarticular bone microstructure and erosions using high-resolution peripheral quantitative computed tomography in rheumatoid arthritis. Clin Exp Rheumatol.

[28] Grech L, Borg K, Borg J (2022). Novel therapies in β-thalassaemia. Br J Clin Pharmacol.

[29] Richard F, van Lier JJ, Roubert B (2020). Oral ferroportin inhibitor VIT-2763: first-in-human, phase 1 study in healthy volunteers. Am J Hematol.

[30] Tjong W, Kazakia GJ, Burghardt AJ, Majumdar S (2012). The effect of voxel size on high-resolution peripheral computed tomography measurements of trabecular and cortical bone microstructure. Med Phys.

[31] Pialat JB, Burghardt AJ, Sode M (2012). Visual grading of motion induced image degradation in high resolution peripheral computed tomography: impact of image quality on measures of bone density and micro-architecture. Bone.

[32] Mikolajewicz N, Bishop N, Burghardt AJ (2020). HR-pQCT Measures of bone microarchitecture predict fracture: systematic review and meta-analysis. J Bone Miner Res.

[33] Frenzen K, Schäfer C, Keyßer G (2013). Erosive and inflammatory joint changes in hereditary hemochromatosis arthropathy detected by low-field magnetic resonance imaging. Rheumatol Int.

[34] Stok KS, Burghardt AJ, For the Spectra Collaboration (2020). Consensus approach for 3D joint space width of metacarpophalangeal joints of rheumatoid arthritis patients using high-resolution peripheral quantitative computed tomography. Quant Imaging Med Surg.

[35] Jensen PS (1976). Hemochromatosis: a disease often silent but not invisible. AJR Am J Roentgenol.

[36] Dejaco C, Stadlmayr A, Duftner C (2017). Ultrasound verified inflammation and structural damage in patients with hereditary haemochromatosis-related arthropathy. Arthritis Res Ther.

[37] Simão M, Cancela ML (2021). Musculoskeletal complications associated with pathological iron toxicity and its molecular mechanisms. Biochem Soc Trans.

[38] Heiland GR, Aigner E, Dallos T (2010). Synovial immunopathology in haemochromatosis arthropathy. Ann Rheum Dis.

[39] Askari AD, Muir WA, Rosner IA (1983). Arthritis of hemochromatosis. Clinical spectrum, relation to histocompatibility antigens, and effectiveness of early phlebotomy. Am J Med.

[40] Husar-Memmer E, Stadlmayr A, Datz C, Zwerina J (2014). HFE-related hemochromatosis: an update for the rheumatologist. Curr Rheumatol Rep.

[41] Karim A, Bajbouj K, Qaisar R (2022). The role of disrupted iron homeostasis in the development and progression of arthropathy. J Orthop Res.

[42] Chehade S, Adams PC (2019). Severe hemochromatosis arthropathy in the absence of iron overload. Hepatology.

[43] Mizoguchi F, Murakami Y, Saito T (2013). miR-31 controls osteoclast formation and bone resorption by targeting RhoA. Arthritis Res Ther.

[44] Stok KS, Burghardt AJ, Boutroy S (2020). Consensus approach for 3D joint space width of metacarpophalangeal joints of rheumatoid arthritis patients using high-resolution peripheral quantitative computed tomography. Quant Imaging Med Surg.

