# Supplementary material for: Analysis of Hand Joint Space Morphology in Women and Men with Hereditary Hemochromatosis
Source: Calcif Tissue Int. 2023 Feb 4;112(4):440–51. doi: 10.1007/s00223-022-01050-3 (PMC10025180; doi:10.1007/s00223-022-01050-3)
Supplement: Supplementary file 1 — Supplementary file1 (DOCX 41 KB) [file 223_2022_1050_MOESM1_ESM.docx]

| **Supplemental Table 1. Patient characteristics of all 24 HH patients and presented by subgroup with and without hand pain at the time of the study visit.** Shown are unadjusted means with standard deviation (SD). Intergroup differences between HH patients with and without joint pain were calculated using Mann-Whitney-U-tests or independent t-tests as appropriate. Significant p-values (p<0.05) are marked in **bold print.** | | | | |
| --- | --- | --- | --- | --- |
|  | **Means ± SD** | | |  |
|  | **All**  **(n=24)** | **With pain**  **(n=17)** | **Without pain**  **(n=3)** | **p value** |
| ***Demographics*** |  |  |  |  |
| Age [years] | 54.7 ± 10.8 | 56.1 ± 10.3 | 45.0 ± 15.4 | 0.124 |
| BMI [kg/m^2^] | 26.6 ± 4.5 | 26.4 ± 4.9 | 27.8 ± 1.8 | 0.303 |
| Gender, female n [%] | 8 [33.3] | 7 [41.2] | 0 [0] |  |
| Time since HH diagnosis [years] | 10.2 ± 9.2 | 11.9 ± 10.2 | 5.0 ± 2.0 | 0.100 |
| Phlebotomies since diagnosis n | 60.6 ± 66.0 | 73.5 ± 73.0 | 24.7 ± 15.5 | 0.189 |
| Annual rate of phlebotomies n | 6.6 ± 11.1 | 7.5 ± 12.8 | 4.7 ± 1.2 | 1.000 |
| ***MCP 2*** |  |  |  |  |
| JSV [mm^3^] | 131.83 ± 32.13 | 122.00 ± 27.51 | 170.34 ± 16.12 | **0.009** |
| JSW [mm] | 1.65 ± 0.34 | 1.60 ±0.36 | 1.87 ± 0.13 | 0.245 |
| JSW.MIN [mm] | 0.67 ± 0.47 | 0.60 ± 0.48 | 0.82 ± 0.70 | 0.546 |
| JSW.MAX [mm] | 2.18 ± 0.30 | 2.14 ± 0.30 | 2.46 ± 0.14 | **0.040** |
| JSW.AS | 9.17 ± 10.35 | 10.19 ± 10.17 | 12.08 ± 17.26 | 0.842 |
| JSW.SD [mm] | 0.33 ± 0.10 | 0.34 ± 0.09 | 0.37 ± 0.19 | 0.799 |
| ***MCP 3*** |  |  |  |  |
| JSV [mm^3^] | 124.51 ± 37.46 | 115.51 ± 36.37 | 159.50 ± 7.80 | 0.116 |
| JSW [mm] | 1.46 ± 0.25 | 1.45 ± 0.24 | 1.74 ± 0.18 | 0.127 |
| JSW.MIN [mm] | 0.63 ± 0.42 | 0.60 ± 0.44 | 1.11 ± 0.06 | 0.118 |
| JSW.MAX [mm] | 2.08 ± 0.28 | 2.10 ± 0.24 | 2.34 ± 0.29 | 0.209 |
| JSW.AS | 7.77 ± 8.39 | 8.50 ± 8.92 | 2.11 ± 0.15 | 0.209 |
| JSW.SD [mm] | 0.33 ± 0.08 | 0.34 ± 0.08 | 0.29 ± 0.07 | 0.327 |
| ***MCP 4*** |  |  |  |  |
| JSV [mm^3^] | 112.08 ± 23.24 | 105.90 ± 21.27 | 133.38 ± 12.75 | **0.048** |
| JSW [mm] | 1.42 ± 0.20 | 1.41 ± 0.22 | 1.46 ± 0.21 | 0.739 |
| JSW.MIN [mm] | 0.75 ± 0.34 | 0.76 ± 0.32 | 0.77 ± 0.47 | 0.975 |
| JSW.MAX [mm] | 1.94 ± 0.22 | 1.93 ± 0.23 | 2.10 ± 0.09 | 0.254 |
| JSW.AS | 4.67 ± 6.47 | 4.18 ± 6.16 | 4.18 ± 3.60 | 0.793 |
| JSW.SD [mm] | 0.29 ± 0.09 | 0.29 ± 0.10 | 0.34 ± 0.10 | 0.359 |

Supplemental Material

**A) Comparison of MCP Joint Space Morphology between HH**

**Patients with and without pain**

**B) Additional Study Information regarding the Subanalysis between**

**HH Patients and Age- and Sex-Matched Controls**

**Controls**

Controls used for all subanalyses in this study were recruited at McCaig Institute for Bone and Joint Health, University of Calgary as part of an ongoing clinical study which was approved by the University of Calgary’s Conjoint Health Research Ethics Board (REB19-0387). The control individuals were recruited via either word of mouth or identified and contacted from a previously populated list of individuals that had consented to be contacted for future research. All patients gave their written consent prior to participation. For the analysis, a total of n = 24 healthy Caucasian individuals were included. Inclusion criteria for control participants required no prior diagnosis of any hand joint problems or inflammatory arthritis prior or within the past 12 months. In addition, individuals were not allowed to have sought out any medication attention on a hand injury and had to be free of any hand symptoms such as current hand pain or swelling within the hands. Additionally, participants had to be older than 18 years of age, not pregnant, and had to be free of any cognitive or physical impairments. Anthropometric information (e.g. height, weight, etc.) was collected on the same day as HR-pQCT scanning.

**HR-pQCT Imaging in Controls**

In all controls, HR-pQCT scans of the MCP joints were acquired using a 2^nd^ generation HR-pQCT scanner (XtremeCT2, SCANCO Medical AG, Brüttisellen, Switzerland). Patients were positioned in a custom position device to reduce motion artefacts, as described by Barnabe et al. [1], and each individual scan was acquired at 68 keV, 1470 𝜇A, 43 ms dwell time, and an isotropic 60.7 𝜇m voxel size. For the MCP joint space morphology analysis of controls, the previously described SPECTRA consensus MCP joint space algorithm was used [2]. Beforehand, automatic contours of the MCP 2 and 3 joint spaces were acquired in HR-pQCT’s native OpenVMS environment (uct_evaluation, v6.6). From these contours, using the SPECTRA consensus MCP joint space algorithm, the standard quantitative metrics of the joint space volume (JSV), joint space width (JSW), the minimal joint space width (JSW.MIN), the maximum JSW (JSW.MAX), the standard deviation of joint space width (JSW.SD) and the joint space width asymmetry (JSW.AS) were computed.

**Reanalysis of HR-pQCT Images of HH Patients using the SPECTRA Consensus MCP Joint Space Algorithm**

In order to be able to compare MCP joint space morphological parameters of HH patients with those of healthy Calgary controls, a subanalysis and reanalyzation of all MCP HR-pQCT images of the HH patients was required using the above described SPECTRA consensus MCP joint space algorithm [2]. This reanalysis of HH HR-pQCT images was necessary as HR-pQCT MCP images of HH patients had been analyzed so far only with the UCSF MCP JSW algorithm as published by Burghard et al. [3] whereas the control group was analyzed with the SPECTRA consensus MCP joint space algorithm which differ slightly in processing. To ensure comparability with control data, the HH-acquired data was therefore reanalyzed with the SPECTRA consensus MCP joint space algorithm [2]. As a previous analysis by Stok et al. [2] had not found significant differences in MCP joint space outcomes between HR-pQCT scanner generations, the data comparability between HH patients and controls was maintained despite the fact that HR-pQCT images for healthy controls were acquired with a 2^nd^ generation HR-pQCT scanner and HR-pQCT images of HH patients had been acquired on a 1^st^ generation HR-pQCT scanner. Results of the final subanalysis comparing MCP joint space morphology in HH patients vs. controls are provided in Tables 3 and 4 of the results section.

**C) Comparison of MCP Joint Space Morphology between Healthy**

**Men and Healthy Women**

To investigate potential sex-related differences in MCP joint space morphology, we computed in an additional subanalysis the intergroup differences in MCP joint space parameters between healthy men and healthy women using the matched control data from the Calgary cohort (Supplemental Table 2).

| **Supplemental Table 2.** Patient characteristics and HR-pQCT-derived joint space parameters of metacarpophalangeal joints (MCP) 2 and 3 shown for healthy men (n = 5) and healthy women (n = 8). Intergroup differences were assessed using independent t-test, if data were normally distributed, or Mann- Whitney-U-test, if data were not normally distributed. Significant p-values (p<0.05) are marked in **bold print**, statistical trends are printed in *italics*.*** | | | |
| --- | --- | --- | --- |
|  | **Means ± SD** | |  |
|  | **Healthy control men**  **(n = 5)** | **Healthy control women**  **(n = 8)** | **p value**  **healthy men vs. healthy women** |
| ***Demographics*** |  |  |  |
| Age [years] | 60.8 ± 15.7 | 58.0 ± 8.2 | 0.686 |
| BMI [kg/m^2^] | 28.0 ± 3.9 | 25.4 ± 3.0 | 0.200 |
| Height (cm) | 173.6 ± 10.5 | 165.9 ±6.5 | 0.131 |
| Gender, female n [%] | 0[0%] | 8 [100%] | 1.000 |
| ***HR-pQCT-derived MCP joint space parameters*** |  |  |  |
| MCP 2 |  |  |  |
| JSV [mm^3^] | 102.5 ± 21.8 | 87.5 ± 11.6 | 0.143 |
| JSW [mm] | 1.79 ± 0.04 | 1.80 ± 0.26 | 0.910 |
| JSW.MIN [mm] | 1.00 ± 0.34 | 1.27 ± 0.37 | 0.204 |
| JSW.MAX [mm] | 2.83 ± 0.10 | 2.78 ± 0.12 | 0.455 |
| JSW.AS | 3.28 ± 1.66 | 2.39 ± 0.83 | 0.107 |
| JSW.SD [mm] | 0.37 ± 0.12 | 0.28 ± 0.07 | 0.124 |
| MCP 3 |  |  |  |
| JSV [mm^3^] | 117.9 ± 13.74 | 87.8 ± 17.10 | **0.007** |
| JSW [mm] | 1.72 ± 0.26 | 1.66 ±0.26 | 0.729 |
| JSW.MIN [mm] | 1.08 ± 0.36 | 1.22 ± 0.21 | 0.385 |
| JSW.MAX [mm] | 2.76 ± 0.11 | 2.65 ± 0.25 | 0.386 |
| JSW.AS | 2.78 ± 0.92 | 2.21 ±0.31 | 0.239 |
| JSW.SD [mm] | 0.34 ± 0.10 | 0.24 ±0.07 | *0.075* |
| HR-pQCT high resolution peripheral quantitative computed tomography  *** All data were analyzed using the SPECTRA consensus MCP joint space algorithm [2] as described in detail in Section  B above. | | | |

**D) References**

1. Barnabe C, Szabo E, Martin L, et al (2013) Quantification of small joint space width, periarticular bone microstructure and erosions using high-resolution peripheral quantitative computed tomography in rheumatoid arthritis. Clin Exp Rheumatol 31:243–250.

2. Stok KS, Burghardt AJ, Boutroy S, et al (2020) Consensus approach for 3D joint space width of metacarpophalangeal joints of rheumatoid arthritis patients using high-resolution peripheral quantitative computed tomography. Quant Imaging Med Surg 10:314–325.

3. Burghardt AJ, Lee CH, Kuo D, et al (2013) Quantitative in vivo HR-pQCT imaging of 3D wrist and metacarpophalangeal joint space width in rheumatoid arthritis. Ann Biomed Eng 41:2553–2564.
